# Cardiac Resynchronization Therapy Using Single Site Left Ventricular Pacing in a Tricuspid Atresia Patient With Left Bundle Branch Block

**DOI:** 10.1016/j.cjcpc.2022.01.004

**Published:** 2022-02-05

**Authors:** Shin Ono, Jan Janoušek, Takeshi Ikegawa, Shun Kawai, Naka Saito, Heima Sakaguchi, Hideaki Ueda

**Affiliations:** a Department of Pediatric Cardiology, Kanagawa Children’s Medical Center, Yokohama, Japan; b Children’s Heart Centre, Second Faculty of Medicine, Charles University in Prague and Motol University Hospital, Prague, Czech Republic; c Department of Pediatric Cardiology, National Cerebral and Cardiovascular Center, Suita, Japan

## Abstract

Cardiac resynchronization therapy (CRT) is typically achieved by pacing both ventricles. However, left ventricular–only pacing has been shown to be noninferior to biventricular pacing in patients with left bundle branch block and normal atrioventricular conduction. However, there is no evidence in favour of CRT with single-site pacing for patients with single-ventricle physiology. In this case, we performed CRT with single-site pacing in a patient with tricuspid atresia and left bundle branch block, enabling successful Fontan completion.

## Résumé

La thérapie de resynchronisation cardiaque (TRC) consiste généralement en une stimulation des deux ventricules. Il a toutefois été montré que la stimulation du ventricule gauche seulement n’est pas inférieure à la stimulation biventriculaire chez les patients présentant un bloc de branche gauche et une conduction auriculoventriculaire normale. Cependant, aucune donnée probante n’appuie la TRC par stimulation d’une seule cavité cardiaque dans le cas d’un cœur univentriculaire. Dans le cas que nous présentons, nous avons eu recours à la TRC par stimulation d’une seule cavité cardiaque chez une patiente présentant une atrésie tricuspidienne et un bloc de branche gauche, ce qui a permis de réaliser l’intervention de Fontan.

A 12-month-old girl (5.6 kg) with tricuspid atresia (TA) and left bundle branch block (LBBB) underwent successful permanent epicardial resynchronization using single-site pacing. She was diagnosed with type 2c TA (TA, ventricular septal defect, and transposition of the great arteries), coarctation of the aorta, and patent left superior vena cava. She underwent aortic arch repair and pulmonary artery banding 7 days after birth, followed by bilateral bidirectional Glenn operation with Damus-Kaye-Stansel anastomosis at the age of 5 months. LBBB occurred after the surgery, posing a risk for adverse effects on the patient’s circulatory status. However, circulatory disruption did not occur, and the patient was discharged from the hospital 3 weeks after operation. After discharge from the hospital, congestive heart failure gradually developed. Unfortunately, this condition could not be controlled despite the administration of diuretics and angiotensin-converting enzyme inhibitors.

**Figure 1:**
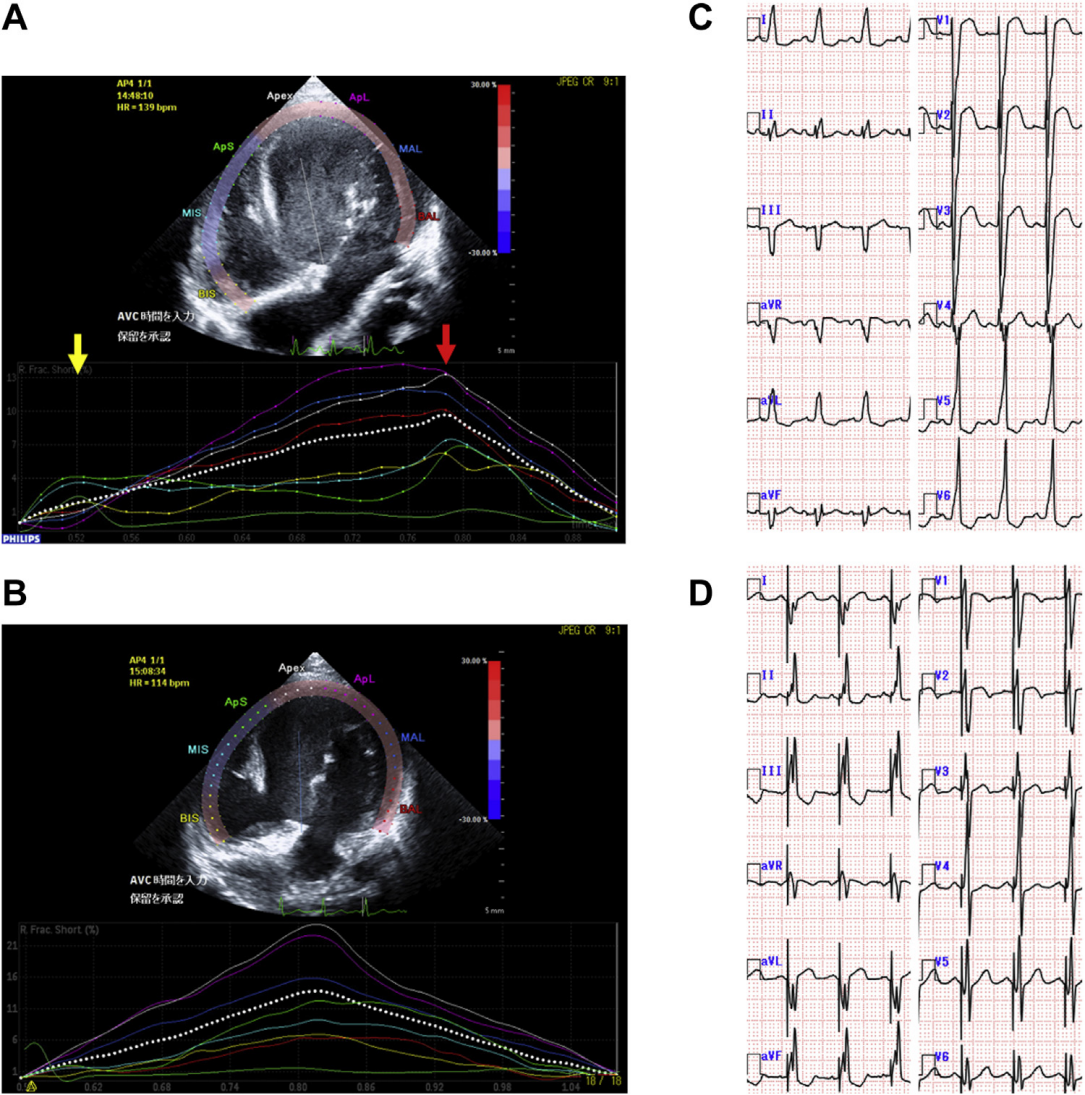
(**A**) Before pacing, early right ventricular (**yellow arrow**) and late left ventricular (**red arrow**) free wall activation with a right-to-left delay of 260 milliseconds was observed. (**B**) Major improvement in interventricular synchrony after left ventricular pacing. (**C**) Native 12-lead electrocardiogram with left bundle branch block. (**D**) Atrial synchronized left ventricular pacing leading to amelioration of the left bundle branch block pattern.

At the age of 8 months, the patient required hospitalization and intravenous inotropic support. Coil embolization of the aortopulmonary collaterals was performed, but it failed to control the heart failure. The serum levels of the N-terminal of the prohormone brain natriuretic peptide (NT-proBNP) ranged 5885-23,720 pg/mL. Ventriculography and echocardiography revealed that the left ventricular (LV) contraction was delayed compared with that of the right ventricle (RV). Electrocardiography revealed LBBB (Fig. 1C), which had not resolved since the operation. These findings suggested that cardiac resynchronization therapy (CRT) was required for this patient. On the basis of physiology of LBBB, we considered that LV pacing in fusion with RV spontaneous activation was sufficient. The patient underwent resynchronization testing in the catheterization laboratory to confirm the effectiveness of LV pacing alone. We compared several hemodynamic indices under 2 conditions, including baseline and LV pacing in fusion with RV spontaneous activation. The LV dp/dt increased from 1156 to 1351 mm Hg/s (PressureWire Certus G8; St. Jude Medical, St. Paul, MN), systolic arterial blood pressure increased from 79 to 94 mm Hg, and QRS duration was shortened from 130 to 109 milliseconds.

**Figure 2:**
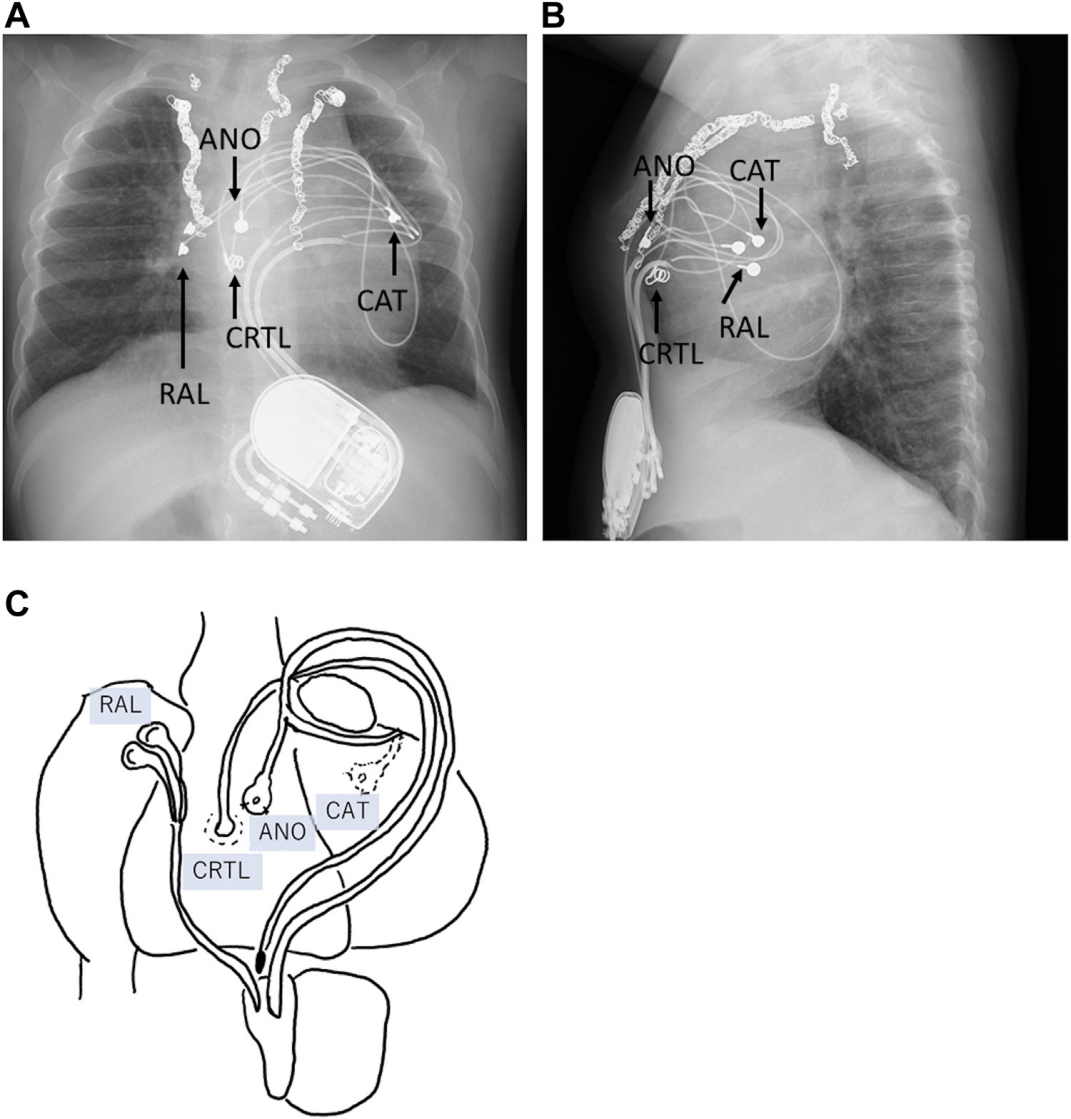
Chest radiograph. (**A**) Anteroposterior and (**B**) lateral view of the pacing system. (**C**) Sites of pacing leads. Atrial pacing leads were placed on the right atrium. The cathode of a ventricular lead was placed on the posterior wall of the left ventricle. The anode was placed on the anterior wall of the right ventricle. An additional lead for cardiac resynchronization therapy was placed on the anterior wall of the right ventricle. ANO, anode of ventricular lead; CAT, cathode of ventricular lead; CRTL, ventricular lead for CRT; RAL, right atrial lead.

One month later, a permanent epicardial dual-chamber pacemaker was implanted. Permanent atrial pacing leads were placed on the right atrium. The cathode of a permanent ventricular lead was sutured on the posterior wall of the left ventricle, whereas the anode was placed on the anterior wall of the RV. The leads were connected to a pacemaker device (Edora 8 DR-T; Biotronik, Berlin, Germany). After the initiation of LV pacing, her blood pressure was increased by 20 mm Hg. The device was implanted in the posterior sheath of the rectus abdominis muscle in the upper abdomen. In addition, we placed another ventricular lead, which was not connected to the pacemaker device, on the anterior wall of the RV for an upgrade to biventricular pacing. We prepared to upgrade to biventricular pacing using anodal pacing or a CRT lead in case of ineffective LV-only pacing (Fig. 2).

The atrioventricular (AV) interval in children exhibits large variability. Therefore, we turned on the negative AV hysteresis function and set the AV delay to be 10 milliseconds shorter when the V lead sensed a self-pulse. The clinical condition improved after pacemaker implantation, whereas the NT-proBNP levels were decreased to 2899 pg/mL. Echocardiography revealed major improvement of interventricular synchrony. Speckle tracking analysis showed that the RV-to-LV delay was almost zero, although the RV-to-LV delay had been 260 milliseconds before resynchronization (Fig. 1, A and B). The 2-dimensional global longitudinal strain of LV also improved from 13.8% to 16.9%. Electrocardiography showed amelioration of the LBBB (Fig. 1D). After another month, the patient was discharged. Total cavopulmonary connection was completed at the age of 2 years. At that time, the NT-proBNP levels were 560 pg/mL.

## Discussion

CRT is a well-established treatment for adult patients with LV systolic dysfunction without congenital heart disease. CRT for patients with single-ventricle (SV) physiology is becoming increasingly common.^1^ However, the evidence regarding the use of CRT in such patients is insufficient. In CRT for patients with SV physiology, we need to take the morphology of the systemic ventricle and mechanism of dyssynchrony into account.^2^ This patient had LV and RV as systemic ventricles. The main ventricle was the LV, but the small RV was not ignorable. Because of the LBBB, the RV contracted first, resulting in interventricular dyssynchrony. To eliminate interventricular dyssynchrony, the LV contraction had to match the RV contraction. Therefore, we considered that LV pacing in fusion with RV spontaneous activation was sufficient. We also considered that the optimal pacing site was the posterior wall of the LV, which is the farthest from the RV.^2^

Currently, there is no evidence in favour of CRT with single-site pacing for patients with SV physiology. However, LV-only pacing has been shown to be noninferior and even superior to biventricular pacing in adult heart failure patients with preserved AV conduction or LBBB.^3^ Regarding other types of congenital heart disease, Kubuš et al.^4^ successfully performed resynchronization with RV-only pacing for a post-tetralogy of Fallot patient with right BBB.

There are other advantages in selecting SV pacing in small children. In paediatric patients with little subcutaneous tissue, there is an advantage in choosing a smaller device in terms of pocket complications associated with generator implantation. In addition, in paediatric patients who will require frequent lead replacement in the future, consideration should be given to reducing the number of leads used.^5^

The risks associated with selecting SV pacing for children are as follows. In children with large variability in heart rate, it is expected that the variability of AV intervals will also be large.^6^ Therefore, the ability to fluctuate the AV delay is necessary to determine the appropriate timing for the fusion of LV pacing with RV self-activation. The negative AV hysteresis function may be a good solution to this problem. This function automatically shortens the AV delay when the ventricular leads sense the self-activation.^7^

We could not determine the reason for LBBB. The patient did not have LBBB before the first surgery. However, she experienced a transient LBBB at the time of her first surgery. The LBBB resolved during the recovery period and did not recur until the second surgery. At the time of the second surgery, persistent LBBB occurred and did not resolve. We could not identify the reasons for the development of LBBB after cardiac surgery, which did not require ventricular resection. Nevertheless, these surgeries are performed under cardiac arrest. Damage of the intraventricular conduction system is a possible complication in this setting.^8^

Although the LBBB occurred immediately after surgery, the patient did not present symptoms of heart failure until discharge. Presumably, the heart failure developed as the patient became more active after discharge from the hospital. It was suspected that LBBB was not the only cause of congestive heart failure. Other undetectable causes, such as cardiomyopathy, may have contributed to the development of LBBB. However, LBBB-induced interventricular dyssynchrony was one of the major causes of the poor circulatory status observed in this patient. Fortunately, we were able to correct the LBBB-induced interventricular dyssynchrony with CRT using single-site ventricular pacing and improve the patient’s circulatory status.

To the best of our knowledge, this is the first reported case of successful CRT using single-site ventricular pacing in an SV physiology patient with BBB and otherwise normal AV conduction.

**Novel Teaching Points**

• Cardiac resynchronization therapy using single-site ventricular pacing may be a good therapeutic option for single-ventricle physiology patients with bundle branch block and otherwise normal atrioventricular conduction.
